# The Physicians Visiting List for 1875

**Published:** 1874-11

**Authors:** 


*The Physician's Visiting List for* 1875. As useful for a dental engagement book as any other in use. Published by Lindsay & Blackiston, Philadelphia.

